# Spatiotemporal Analysis of Influenza Morbidity and Its Association with Climatic and Housing Conditions in Ecuador

**DOI:** 10.1155/2019/6741202

**Published:** 2019-11-23

**Authors:** Andrea Lobato-Cordero, Emmanuelle Quentin, Gina Lobato-Cordero

**Affiliations:** ^1^Instituto de Investigacion Geologico y Energetico (IIGE), Quito, Ecuador; ^2^Instituto Nacional de Investigación en Salud Pública (INSPI), Centro de Investigación EpiSIG, Quito, Ecuador; ^3^Universidade Federal de Uberlândia (UFU), Uberlândia, Brazil

## Abstract

The external environment directly influences human health. However, what happens inside? This work deals with the effect that the interior thermal variables have on the propagation of respiratory diseases and focused on the relation of the temperature and relative humidity inside social housing in the 1040 parishes of Ecuador and the transmission of influenza. On the one hand, historical weather-related variables were used to simulate and estimate the interior conditions, and thresholds on temperature and humidity were determined. On the other hand, the health-related variable was determined by analyzing the statistics corresponding to the influenza and viral pneumonia in 2009 since that year was critical for these diseases; the data were divided by month for each parish. Finally, the correlation of these variables determines the relative importance of the interior conditions on the respiratory health of its inhabitants. The preliminary results indicate that the places with the lowest temperatures and relative humidity could favor the virus transmission. Also, the analysis indicated that respiratory diseases increase in August and October. In this way, it is clear that social housing projects in Ecuador require a study which guarantees not only energy efficiency and sustainability related issues but also the well-being of their inhabitants.

## 1. Introduction

Latin America faced sanitation and hygiene problems during the colonial period since Europeans imported those problems with them. In addition, to exert all their power, the settlers replaced the way of life, food, hygiene practices, and culture of natives by their own customs, which not necessarily were the best for the new world since the environmental conditions are different between America and Europe. For example, Latin American countries that are closest to the Equatorial line do not have four marked seasons. Despite those differences, the housings used in the new colonial cities did not consider the local conditions. Initial approaches to building analysis include overcrowding, ventilation, humidity, lighting, acoustics, and waste disposal [1].

Since the new houses do not follow the practices of the colonial era, new problems appeared. For instance, migration or displacement caused by political, social, and economic problems or natural disasters contributes to overcrowded spaces with restricted areas to live, where the constructions have sanitary deficiencies, promoting the transmission of respiratory or infectious diseases [2]. According to [3], deficient houses are those that do not properly protect from sicknesses, accidents, and even mortal danger.

For this reason, the structure of houses and the conditions in rooms have a direct influence on health [4]. According to the World Health Organization (WHO), one of the housing functions is the protection against transmissible sicknesses. It has been demonstrated that overcrowding in combination with conditions of poverty and insufficient access to utilities increases the rate of transmitted diseases through the air such as influenza and common cold [3]. An overcrowding study indicates that different levels exist: the room-level (including “bed crowding”) and the building-level crowding and “poses serious direct and indirect health risks to all segments of the population, particularly the elderly and young children” [5].

This lack of proper living conditions directly affects the indoor air quality [6]. The influence of housings on the health of its inhabitants has been directly correlated [7]. In Latin America and Caribbean, approximately 34 million families [8] live without access to clean water, sewerage, with nonsuitable floors, or insufficient space. Noise, space, and light in interior spaces must be considered to promote the well-being of the people who live inside [9]. “Indoor environment, and especially housings, represents one of the major health determinants, and a clear and updated regulatory system is a key factor to ensure Public Health Protection” [10]. Physical conditions in social housings could potentially increase the risk. The selection of construction materials responds to cultural, economics, and availability patterns; therefore, the use of inadequate construction materials also affects the housing conditions and the occupants' health [11]. For example, researchers in [12] demonstrated the link between people's health and security with the materials which were used in the construction. “The main health outcome shown to be related to housing is that of respiratory health, measured by the presence of respiratory disease or by lung function” [7]. This kind of housing solves principally a space problem but does not provide proper indoor thermal conditions or high air quality; moreover, these parameters are affected by the high number of inhabitants.

In the same way, the exterior environment and the interior microclimate of inhabited spaces interact with the construction materials and influence the interior [13]. Variables such as air temperature, radiant average temperature, relative humidity, air speed, clothing, and physical activity influence the hygrothermal comfort [14]. It is important to consider the impact of weather on the performance of buildings [15] and how changes in climate patterns affect human health [16]. In addition, certain climatic conditions facilitate the proliferation of microorganisms such as bacteria and fungus on ecofriendly materials [17]. For instance, the increase in the relative humidity in the air affects the humidity content of permeable materials such as wood, paper, carpet, and food allowing mildew to spread, which reproduces trough spores transported by air [18], generating respiratory problems [2]. There is evidence that links the humidity in the interior and allergies and respiratory diseases [19]. Cases of children allergies in Seoul have been associated with the characteristics of buildings. Also, some survey results showed that respiratory diseases are associated with a higher level of humidity produced by the material used in the construction [20]. A study performed at homes in Britain concludes “… at extremely low temperatures cardiovascular problems occur and the risk of hypothermia rises. Less severe cold conditions encourage condensation, and parents report more respiratory problems in their children if they live in damp houses” [9].

A high percentage of buildings are vulnerable to climatic conditions, mainly to the temperature changes depending on the time of the day [21]. These hourly changes influence the relationship between the occupants' comfort and their health [22], mainly with those who spend their indoor time mainly at night between 11 PM and 5 AM in bad conditions [23], or among those who maintain thermal comfort during the day, as in residences [24]. In consequence, the interior environment of a house is one of the aspects that can be improved with adequate environmental policies and regulations. Examples such as the correct application of the best construction practices are key to “… better understanding of the interactions between specific housing and building conditions with physical and mental health outcomes” [25].

On the contrary, influenza is “… an acute respiratory infectious disease related to climate and seasonal clustering cycles” [26], which has caused global epidemics and pandemics [27], due to its rapid transmission. “In temperate climates, seasonal epidemics occur mainly during winter, while in tropical regions, influenza may occur throughout the year, causing outbreaks more irregularly” [26]. Most influenza infections are spread by droplets of several microns in diameter that contain the virus and are expelled during coughing and sneezing [27]; therefore, “the airborne route is a potentially important transmission pathway for influenza in indoor environments, especially in unventilated conditions” [28]. It supposed that most cases of influenza are transmitted by aerosol “suspensions in air (or in a gas) of solid or liquid particles, small enough that they remain airborne for prolonged periods because of their low settling velocity” [29]. And “when an infected person coughs or sneezes, droplets containing viruses (infectious droplets) are dispersed into the air and can spread up to one meter, and infect people in close proximity who breathe these droplets in” [26], in this case inside a housing during the night. A study about the transmission of influenza A(H1N1)pdm09 in the pandemic and postpandemic seasons in a house shows that the probability of virus transmission varies according to the weather station, and that people who shared a bedroom were at a higher risk of presenting influenza-like illness, being the children the most affected [30]. The percentage of influenza contagion is very high worldwide so that the development of symptomatic influenza A or B each year in children represents about 20% and 5% of adults [27].

It is important to consider that people live in social housing are mostly kids and elders [31] spend more time inside houses that could affect their health [32]. To prevent health issues related to inadequate living conditions, researchers in [33] state that temperature should be 18–24°C and relative humidity is 20–70% (ideal relative humidity is 40–50%). Although influenza is a common illness, it can condition the daily activities of an affected person. In addition, new influenza viruses which threaten the human life have been identified, such as the one that caused the influenza pandemic [33] in 2009 [34]. The World Health Organization (WHO) reported that “… H5N1 influenza virus has caused the infection of 650 people and the death of 386 since 2003” [35]. The routes of transmission of the influenza virus in humans have not been defined, but evidence is consistent with bird-to-human, possibly environment-to-human, and, in limited cases, human-to-human transmission human influenza A (H5N1) infections are limited [36].

The rapid transmission in crowded areas including schools and nursing homes has been confirmed by the WHO. The main objective of this study is to raise the hypothesis about relationship between the thermal conditions inside social housing projects and respiratory diseases in Ecuador. Specifically, the relation between influenza virus transmission, temperature, and relative humidity (RH), as potential conditions for the increasing virus transmission, is analyzed. For the analysis, it was considered the temperature information and relative humidity stimulates virus transmission (A/Panama/2007/99 virus), based on results of experiments done on guinea pigs in controlled spaces and inoculated with a human stain of the virus and healthy guinea pigs.

In Ecuador, local governments have between their competences to promote “… safe and healthy habitat and guarantee the right to have a dwelling” [37], supported by laws and norms proposed by the central government. The minimum conditions in housings are determined by Ley Orgánica de Ordenamiento Territorial, Uso y Gestión de Suelo (law to regulate the use of the land) which establishes the following: (a) to have access to clean water, electricity, and sewage system in working conditions; (b) finished kitchen and bathroom; (c) elevators when housings are buildings if they are required in the national and local construction codes; (d) completely built communal areas in buildings and blocks of buildings” [38]. In the same manner, housings considered to be social housing that are “…assigned to priority attention human groups, and population in situations of poverty or vulnerability must be adequate and DIGNA” [39]. The National Development Plan 2017–2021 has an indicator that measures the deficit in the housing quality [40], which considers the type of housing, the material in the floors, walls, and roof, and the state of materials; however, the same plan states that “… it is relevant to incorporate new indicators that allow to evaluate the quality of the living conditions and the access to utilities” [39]. Currently, there is a code focused on the thermal behavior and energy efficiency in new housing that consider the local weather [41], aligned with the Government's housing policies. Finally, a plan to improve the living conditions of families in extreme poverty and vulnerable situation has being executed “to guarantee the right of people to have a safe and healthy environment and an appropriate and worthy housing, independently of their social and economic situation.”

The compliance with the code and the use of improved construction materials will allow to solve short- and long-term overcrowding problems [5], in addition to promote the health and well-being of people [7]. In this sense, a house renovation can improve the health conditions of his inhabitants [42] so that this task is not only a construction problem but also a public health problem [7].

A previous study introduced the topic of spatially relating infectious diseases with climatic and housing conditions in Ecuador without statistics evaluation of the link [43]. This work is a first step in the research related to the exposure of this disease and its relation with the incidence, prevalence, mortality, or any other factor of interest for Ecuadorians. The results will be very useful to identify the conditions that affect the quality of life of the people who live in these houses, considering that there is a 33.1% housing deficit in the country [44]. Furthermore, it will allow to identify habitability parameters that must be articulated with energy efficiency standards, constructive characteristics, and other factors which affect the interior conditions of inhabited spaces.

## 2. Materials and Methods

For this work, an epidemiologic approach has been used, but as the data available were aggregated by geographic unit, only the ecologic type of study can be applied, adapting the quantitative method of relative risk estimation to it, in order to relate possible determinants to spatial distribution of disease [45]. Temperatures were assumed between 5°C and 20°C and relative humidity of 20%, 35%, 50%, 65%, and 80% [46]. These parameters were applied to a digital model of social housing defined by Ministerio de Desarollo Urbano y Vivienda (Miduvi) (Ministry of Urban Development and Housing) in Ecuador. This typical house is unique for all Ecuadorians, and no adaptation to specific regions has been considered for economical reasons. The behavior of indoor conditions of temperature and relative humidity was simulated in DesignBuilder software. This social house model has been established for the whole country including urban and rural zones, using the same architectural design, dimensions, and materials. However, the weather conditions in the 1040 parishes in continental Ecuador are different. The rate in each one of the 1040 parishes was calculated using hospital records from Instituto Nacional de Estadística y Censos (INEC) (National Institute of Statistics and Census) for cases coded from J09 to J12, according to the World Health Organization International Statistical Classification of Diseases and Related Health Problems, 10th Revision (IDC-10) and population totals. The housing models for the simulations are the same for each parish; however, its behavior changes according to the climatic conditions that will generate internal and external temperature and relative humidity values. In spite of relative climatic stability, there is a variation in the temperature between day and night; specifically, the temperature at sunset is decreasing and reaches its lowest values at night, when the house is more occupied.

On the contrary, cases from hospital records were processed and mapped by parish, selecting the International Disease Codes from J09 to J12 (influenza and viral pneumonia), in order to verify if some relations exist. In relation to the risk factors for influenza virus transmission by contagion, the threshold encountered in one of the few experimental studies on the subject [47] was used to determine the temperature and relative humidity limits inside the house during occupation hours.

**Figure 1:**
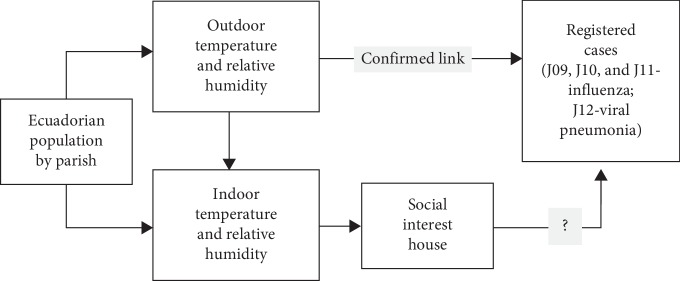
Schematic diagram of relationship between the thermal conditions inside social housing projects and the registered cases of J09, J10, J11-influenza, and J12-viral pneumonia assuming that “the relationship between climate variability and illness is described as the net effect of climate variability on illness incidence based on individuals' exposure level” [48].

The conditions of temperature and relative humidity indoor a social interest house, and its hourly variation during one year has been estimated for each parish [47], using the same values for architectural design, materials, and occupation time, but exposing the house to different climatic conditions (Figure 1).

### 2.1. Zones Classification

The spatial division of Ecuador was identified by a six-digit primary code that represents each parish as a political-administrative unit [47]. This type of coverage is a polygonal vector that has the same characteristics in each unit, from which a centroid was calculated for each register with the objective of substituting every polygon for its representative spot. This characteristic allows for inquiries and links with the databases about houses and population census made by INEC, as well as with the health databases compiled by the INEC from registers of the Public Health Ministry.

The source for the elevation digital model is the NASA Shuttle Radar Topography Mission (SRTM) at a resolution of one arc-second (approximately 30 meters) available for most of the land surfaces that lay between 60°N and 54°S [49]. Once imported in a Geographic Information System (GIS), the elevation of the centroid of each parish can be extracted from this raster layer, and the weather conditions related to those points can be estimated. For this analysis, information about the 1040 parishes of the country was used.

### 2.2. Definition of the Weather Conditions

To define weather conditions, we generated meteorological information in EPW format with hourly data, through Meteonorm software for each parish. Because of the large quantity of iterations and combinations, it was necessary to use an automatization process. Once the working environment was defined, we used a virtual box to assemble all the tools over a virtual machine (VM), Mouse Recorder Pro (MRP), which was used to record the sequence that Met provides to obtain the files. MRP calendar allowed scheduling the task to be performed at a specific hour; besides, it was used to repeat the cycle as many times as needed. Once the configuration was done, the VM was turned off and its status was saved. The current VM was cloned 10 times; hence, for gaining velocity and saving space, a cloned cycle was linked to the original VM.

The reports created in the VM were configured to simulate them with different data; thus, they were distributed between them. The start mode without windows was used to keep running the VMs simultaneously. When the simulation on Met was finished, all the results were grouped and then the VMs were deleted. For validating the results, a script was programmed in Python with the aim of verifying that the coordinates and the specified height per each block were the same in the weather files and also in the ones that were used to obtain the results.

### 2.3. Respiratory Diseases Registry Definition

The database used to obtain the influenza morbidity was the hospital registry made available by the INEC. The cases of 2009, considering the code J09 to J12, have been summed by parish, which is the smallest political-administrative limit available in the database. To obtain the disease rates, the population used as the denominator was extracted from the 2010 INEC census since one-year difference will not affect the relative value between parishes.

The disease group selected included the J09-influenza, related to the zoonotic or pandemic influenza virus, the J10-influenza related to the seasonal influenza virus, the J11-influenza, which is a not identified virus, and the J12-viral pneumonia, which has not been classified elsewhere. The last one has been included since its propagation is similar to the influenza (other pneumonia have bacterial cause).

### 2.4. Definition of the Indoor Conditions

The data of indoor conditions were obtained using simulations of the thermal behavior of house models. EnergyPlus linked with the DesignBuilder (DB) environment was used to perform simulations with virtual building models. EnergyPlus is capable of performing simulations with “time steps of less than an hour, modular systems and plant integrated with heat balance-based zone simulation, multizone air flow, thermal comfort, and photovoltaic systems” [50]. Also, “it provides a range of environmental performance data such as energy consumption, carbon emissions, comfort conditions, daylight illuminance, maximum summertime temperatures, and HVAC component sizes” [50]. In addition, the software has a range of input tabs and database including geometric data of the model, templates of materials, occupancy [51], weather files, natural ventilation, advanced solar shading, and internal comfort [52].

The weather files needed to be converted in EPW format before using them. This task was performed only once. The input data for the DB software simulate and assess the indoor thermal behavior in each parish were as follows.

**Figure 2:**
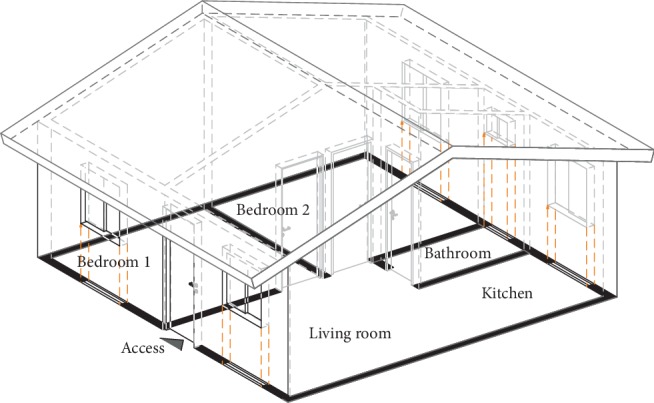
Redrawing of the marginal urban and rural housing model [53].

Housing model: the house has 36 m^2^ of construction area and has two bedrooms and one bathroom, living room, and kitchen in a single area. All these areas are distributed in one floor and covered by an inclined roof (Figure 2).

#### 2.4.1. Occupation Schedule

The occupancy index is the relation between the occupancy density and the occupied surface area of the building (Table 1).

#### 2.4.2. Air Tightness of the Houses

Air tightness is defined in accordance to DesignBuilder classification, considering that the model house has high permeability. The parameters used were the fissures standardized according to the length of the perimeter of the gaps: internal and external windows, grids, and doors. The fissures on the roof, walls, and floor were modelled according to the average porosity of one standardized fissures, based on the surface area (Table 2).

#### 2.4.3. Materials

The influence of the materials on the internal conditions of houses has been demonstrated. However, this kind of houses in Ecuador uses the same materials in every zone where they are built (Table 3).

Starting with these data, the simulation process per each parish was automated again. Using the MRP, the task was saved to be executed to simulate the housing behavior with DB. The VM was turned off to save its status; on top of this, ten-linked VM clones were created. These VMs needed to be configured to run simulations with different data. Once the simulation process finished, all output files were unified, the VMs were deleted, and the results were validated using a python script.

The next step was to save the generated information in a dimensional database, which can be worked jointly and not as individual files. To perform this task, it was necessary to design a relational database. The Pentaho Data Integration (PDI) tool of the Pentaho Suite of business intelligence was used to upload all the information in the database.

### 2.5. Statistical Analysis

To evaluate the association between the disease and the exposure to specific climatic and housing conditions, the relative risk (RR), Equation (1), was used, extending its application to ecological or correlation study. This indicator was applied for each month. For this, Table 4 was constructed by counting how many parishes with high rate of influenza also have, either an indoor temperature over 20°C and RH greater than 35% (value “a”), temperature lower than 20°C and RH under 35% (value “b”); on the contrary, the parishes with low rate of influenza are also divided between housing with an indoor temperature over 20°C and RH greater than 35% (value “c”) and housing with an indoor temperature below 20°C and RH lower than 35% (value “d”):

(1) $$\begin{array}{c} \text{RR}=\frac{a/\left(a+b\right)}{b/\left(b+d\right)}, \end{array}$$

(2) $$\begin{array}{c} {\text{CI}}_{95\%}\text{RR}=\exp\left(\ln\text{RR}\pm 1.96\sqrt{\frac{1-\left(a/\left(a+b\right)\right)}{a}+\frac{1-\left(c/\left(c+d\right)\right)}{c}}\right). \end{array}$$

A relative risk over 2 indicates a positive association between exposure and disease; meanwhile, a value lower than 2 indicates that the exposure could be considered as a protection against the disease. A value of one does an inconclusive result [54]. There is a need to be careful with the interpretation of the relative risk as applied not on individual cases but on aggregated values.

The amplitude of the RR is calculated by obtaining the confidence interval (CI), in a range between the upper interval (UI) and lower interval (LI) (Equation (2)) [55]. RR has a statistically significant association if the result includes the value of 1 [56].

## 3. Results and Discussion

A correlation of 0.96 between external and internal temperature was observed in every analyzed parish, with an average temperature difference of 4.38°C.

In a normal year, the annual mean temperature ranges between 17 and 20°C. Nevertheless, those values vary according to their proximity to the Pacific coast, the Highlands region, or the Amazonia.

**Figure 3:**
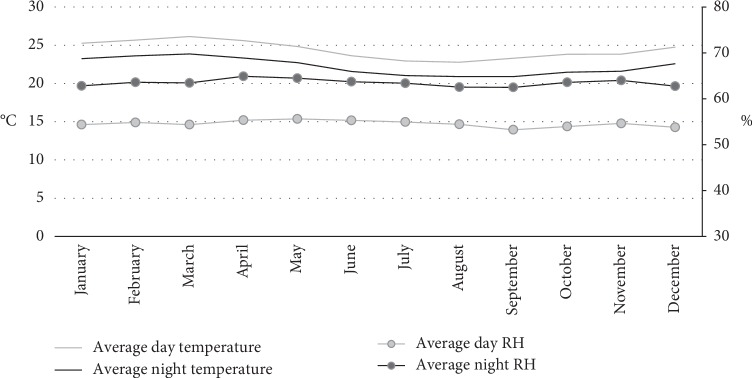
Average indoor temperature and relative humidity of the typical house during the day and during the night.

Because of the equatorial location of the country, the temperature during the year in Ecuador is relatively constant, with a difference between day and night of 3°C, approximately (Figure 3).

**Figure 4:**
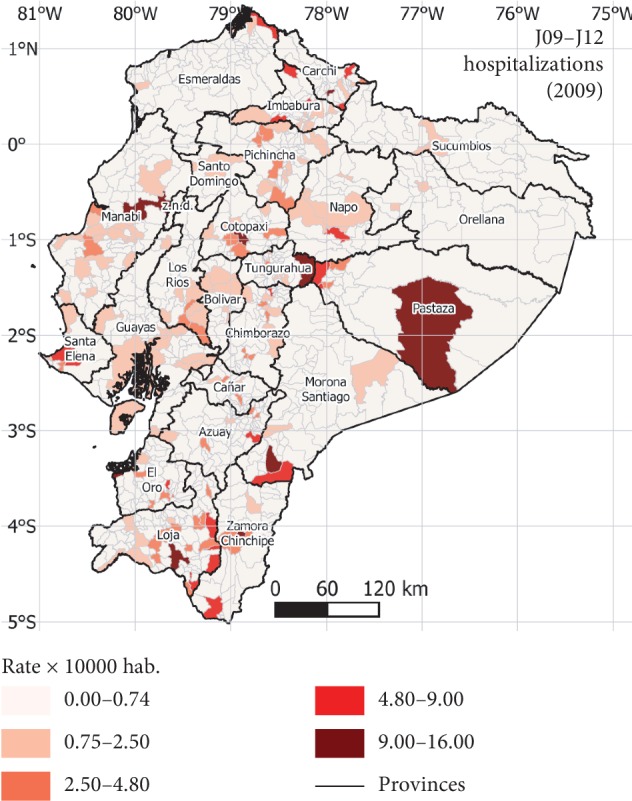
Rates of J09–J12 per parish in Ecuador.

The indoor temperature and relative humidity for each one of the 1040 parishes were extracted for the simulation results. Using the control thresholds of 20°C for temperature and 35% for relative humidity [57], the simulated data allow to discriminate between parishes with exposure to adequate conditions for influenza transmission from parishes with less exposure. The map in Figure 4 presents the disease rates, and no spatial pattern can be clearly identified at this stage.

**Figure 5:**
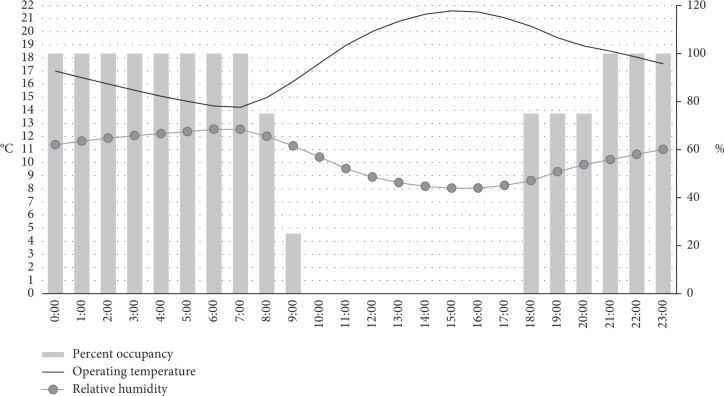
Annual average occupancy, temperature, and relative humidity by hour for the Pillaro parish.

The indoor thermal behavior significantly varied between day to night; consequently, the hourly variation within a day is an important parameter. The period between 18 : 00 PM and 08 : 00 AM has been selected for the analysis since it coincides with an occupancy rate between 75% and 100%.  Figure 5 illustrates the fluctuation simulated for a parish located in the highlands.

The monthly results of relative risk, taking into account the 1040 parishes, are not constant and present values statistically significant in August and October (Table 5). For those two months, the risk of having people who suffer from respiratory diseases in parish is higher if the temperature and relative humidity are lower than the threshold values.

Considering the theory of multicausality in diseases [58], there could be a combined interaction between the indoor temperature and relative humidity that impacts the disease occurrence. These would be factors that cause the disease, which, when avoided, contribute to prevention [56]. In this study, cases appear significantly during the months of August and October.

At a country level, in 2009, the results confirm the hypothesis that low indoor temperature and low relative humidity are significant factors that increase the risk of transmission of influenza, particularly in August and October.

Despite the climatic conditions being relatively stable during the year in Ecuador, hourly oscillations impact the temperature and relative humidity inside the house. These characteristics are similar in territories located in the equatorial zone where habitability conditions are assumed and, generally, the buildings are not adapted to the environment or adopt inappropriate standards.

The followed methodology allowed a preliminary analysis that suggests what the next steps in this research should be. A recent document was published by the World Health Organization [59] that one of the indicators used to determine the severity of a possible influenza pandemic is the transmissibility of an influenza virus, revealing the importance of finding a method to evaluate the dynamics of the spread of the disease.

The results can be refined if more reliable statistics related to respiratory diseases are obtained, or if more detailed climatic data are available. It is important to consider that the indoor quality environment affects the inhabitants, mainly in houses where people spend a significant amount of time resting and recovering from their daily activities.

Climatic parameters that could affect the well-being of the people must be included when energy efficiency standards are updated. Currently, in Ecuador, Chapter 13th Energy Efficiency in residential buildings of the Ecuadorian Building Standard (Norma Ecuatoriana de la Construction (NEC)) is being driven by Miduvi. However, temperature and relative humidity indoor defined in standards in energy efficiency would be consider the health condition of the occupants since it could also have an impact on public health.

In Ecuador, the temperature inside the social housing is correlated with the outside temperature. However, the temperature difference between day and night can be up to 10°C, approximately. In this sense, the variation of temperature in one day can affect the health of the occupants of the edifications and much more to those of social housing. Statistically, these conditions could contribute to influenza virus contagion, particularly during August and October, as a consequence of the correlation between the indoor and outdoor temperatures during the periods of maximum occupancy.

It is important to analyze the different factors of housing which can contribute to improve the conditions inside. One of them, thermal insulation is not used in enclosures of space. This factor can affect the stability of conditions inside the house and consequently impact on occupants' health, but this relationship should be validated with real-field measurements.

## 4. Conclusions

The temperature and relative humidity conditions inside the homes are related with the outside climate, but these can change depending on the characteristics of the materials of housing envelope, infiltration, percentage of glazing, height of the roof, and among many other construction features that must be appropriate to the climate. The indoor microclimate varies, being able to increase or decrease the transmission of diseases among its inhabitants. This work found that a part of the housings that do not comply with temperature and relative humidity standards in the interior are built with inappropriate materials so that the well-being of their inhabitants is affected. In addition, an increased rate of influenza spreading, in combination with overcrowding, converts these housings in origin of spreading diseases.

In addition, the analysis shows that the socioeconomic status of people and an absence of laws that regulate the hygiene in the housings (social housing mainly) will negatively impact the health and economic situation of Ecuadorians. This fact was already demonstrated and addressed since some codes and laws has been stablished; nevertheless, these laws should be updated to account for specific local weather conditions and the use of modern materials.

This ecological study makes it possible to prioritize study areas in which housings are affected or could be affected depending on climatic conditions and the characteristics of the housings.

## Figures and Tables

**Table 1:** Weekdays occupation of housing.

Hours				Occupation
00 : 00	—	07 : 00	=	1.00
07 : 00	—	08 : 00	=	0.75
08 : 00	—	09 : 00	=	0.25
09 : 00	—	17 : 00	=	0.00
17 : 00	—	20 : 00	=	0.75
20 : 00	—	24 : 00	=	1.00

**Table 2:** Value of the air tightness in the housing.

Category	Flow (kg/s·m^2^@1 Pa)		Coefficient flow exponent	
	Outdoor	Indoor	Outdoor	Indoor
Windows	0.0030	0.003	0.60	0.60
Doors	0.0030	0.003	0.66	0.60
Grids	0.0400	0.020	0.66	0.60
Walls	0.0004	0.019	0.70	0.75
Floors	0.0020	0.003	1.00	0.70
Roofs	0.0002	—	0.70	—

**Table 3:** Materials in the housing coverage.

	Floor	Walls	Windows	Roof
Materials	Cement	Brick	Simple clear glass	Clay tile-gypsum
U (W/m^2^K)	1.919	2.510	5.77	6.060

**Table 4:** Input values for relative risk calculus.

		Exposition	
		Parishes	Parishes
Parishes		Over 20°C and 35% RH	Below 20°C and 35% RH
Disease	With cases	a	c
	Without cases	b	d
		*a* *+* *b*	*c* *+* *d*

**Table 5:** Input values for relative risk calculus.

	January	February	March	April	May	June	July	August	September	October	November	December
RR	1.02	0.35	0.46	0.87	0.60	2.96	1.22	4.56	3.60	3.19	2.75	1.88
CI 95%	0.73 1.40	0.07 1.69	0.12 1.74	0.28 2.71	0.18 1.98	0.79 11.09	0.45 3.34	1.31 15.89	0.75 17.23	1.04 9.83	0.87 0.72	0.74 8.72

## Data Availability

The indoor hourly results of temperature and relative humidity used to support the findings of this study are available from the corresponding author upon request.
